# Pathological changes are associated with shifts in the employment of synonymous codons at the transcriptome level

**DOI:** 10.1186/s12864-019-5921-9

**Published:** 2019-07-09

**Authors:** Eugenio F. Fornasiero, Silvio O. Rizzoli

**Affiliations:** 1 Department of Neuro- and Sensory Physiology, University Medical Center Göttingen, 37073 Göttingen, Germany; 2 Center for Biostructural Imaging of Neurodegeneration (BIN), 37075 Göttingen, Germany

**Keywords:** Codon usage, mRNA abundance, Molecular diagnostics, Pathology meta-analysis

## Abstract

**Background:**

The usage of different synonymous codons reflects the genome organization and has been connected to parameters such as mRNA abundance and protein folding. It is also been established that mutations targeting specific synonymous codons can trigger disease.

**Results:**

We performed a systematic meta-analysis of transcriptome results from 75 datasets representing 40 pathologies. We found that a subset of codons was preferentially employed in abundant transcripts, while other codons were preferentially found in low-abundance transcripts. By comparing control and pathological transcriptomes, we observed a shift in the employment of synonymous codons for every analyzed disease. For example, cancerous tissue employed preferentially A- or U-ending codons, shifting from G- or C-ending codons, which were preferred by control tissues. This analysis was able to discriminate patients and controls with high specificity and sensitivity.

**Conclusions:**

Here we show that the employment of specific synonymous codons, quantified at the whole transcriptome level, changes profoundly in many diseases. We propose that the changes in codon employment offer a novel perspective for disease studies, and could be used to design new diagnostic tools.

**Electronic supplementary material:**

The online version of this article (10.1186/s12864-019-5921-9) contains supplementary material, which is available to authorized users.

## Background

The genetic code comprises 64 combinations of codons, 61 of which encode for 20 different amino acids. With the exception of methionine and tryptophan, all amino acids are encoded by more than one synonymous codon. The typical amino acid is encoded by an equal number of codons ending in guanine or cytosine (GC3) and in adenine or uracil (AU3). Although it is not entirely understood how the preferential use of GC3 or AU3 codons arises [1, 2], it is becoming clear that this aspect influences several aspects of cell biology including genome architecture [3], transcription [4], mRNA abundance and stability [5, 6], translation efficiency [7, 8], protein structure [9] and eventually protein expression levels and protein stability [10–12]. Since all these processes concur to the maintenance of protein homeostasis [13], with the purpose of optimizing the efficiency of gene architectures [14], codon employment can be considered as a common regulatory mechanism that orchestrates cell and tissue performance [9].

All of these effects have come to be known under the term “codon usage”, although this has classically been employed to describe the codon effects on translation. Codon usage has also been connected to disease, for more than a decade, typically through changes on gene and/or protein function that are induced by mutations that change one specific codon for a synonymous one [15]. In addition, more recent observations suggest that in certain pathophysiological states some synonymous codons become preferentially used, while others are preferentially neglected, as observed during cellular stress or in neoplastic transformations in humans [16, 17]. Such changes in codon usage could be readily measured by differential expression analysis workflows that have been previously applied to yeast and nematodes [18], based on the many different human transcriptomic databases that are currently available [19].

Thus, one could test relatively easily whether specific codons are preferentially employed in certain disease states. To our knowledge, this idea is largely unexplored for human disease and only few examples are present in the literature [17, 20, 21]. We decided to systematically apply it to 40 common health disorders. We first found that human transcripts (mRNAs) show a well-defined bimodal distribution on the basis of the third nucleotide, with peaks defined by AU3-rich transcripts and GC3-rich transcripts. We found that the AU3-rich genes were involved in proliferation, while the GC3-rich genes were closely linked to cell differentiation and specialized function. This codon separation between proliferation and differentiation genes was more prominent in mammals than in other animals, and was strongest for human. This separation suggested that diseases that trigger proliferation, such as cancer, should increase the employment of AU3 codons in the transcriptome, while reducing the GC3 employment. To test this, we analyzed the codon employment in several pathologies, including myopathies, muscle dystrophies, cancer, several infections, and other common health disorders. The codon employment shift we predicted took indeed place in all diseases, either by favoring the employment of AU3 codons or by favoring the CG3 codons. The codon employment shift was strong enough to serve as a diagnostic tool for several studies.

## Results

### The distribution of GC3 and AU3 codons in transcripts from humans and other organisms

**Fig. 1:**
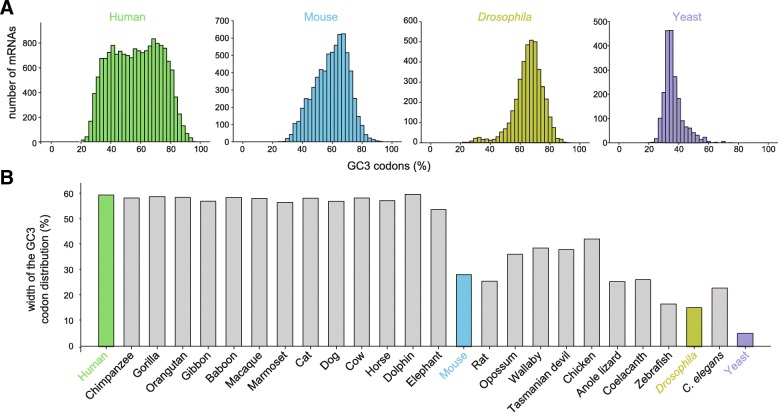
Frequency distribution of GC3 codons in mRNAs for human, mouse, *Drosophila* and yeast, and width of the distribution for several organisms. **a**. Frequency distributions of GC3 codons. We would like to point out that the distribution in human is wide, from genes composed of mainly GC3 codons to genes composed mainly of AU3 codons (with proportionally low amounts of GC3 codons). This is not the case for every organism. **b**. Width of the GC3 codon distribution, measured as full width at half maximum of histograms such as those in **a**, plotted for each organism

To understand if the nature of the third nucleotide can be subject to regulatory mechanisms, we initially took into consideration some quantitative aspects of GC3 and AU3 codons, as their frequency across different transcripts. For this purpose, we analyzed 16,497 reviewed protein coding regions from the annotated human genome (GRCh38) and we calculated the average GC3 composition of these transcripts. Human transcripts show a wide range of different average GC3 percentages (from ~ 20% to ~ 98% with a median of 57.5%; Fig. 1a leftmost panel). Overall, we observed a broad distribution, with two peaks at around 70% GC3 and 30–40% GC3, indicating that some transcripts are made mostly from AU3 codons, while others consist mostly of GC3 codons. While these observations seem trivial, it is worth pointing out that not all organisms make such a broad use of GC3 and AU3 codons. For example, the mouse and the *Drosophila* codon sequences are strongly biased towards GC3 codons, while yeast transcripts use mainly AU3 codons (Fig. 1a, remaining panels).

A more extensive observation of the width of the GC3 codon distribution (calculated as full width at half maximum), shows a wide range of values among different organisms (Fig. 1b). The width is extremely limited in yeast, but is more extensive in larger vertebrates, and in particular in placental mammals. This observation is particularly interesting, since a wider spread would provide a larger dynamic range for regulatory mechanisms that might rely on codon employment for cellular modulation (see below).

### GC3 codons tend to find themselves in the same genes, and they tend to avoid AU3 codons

**Fig. 2:**
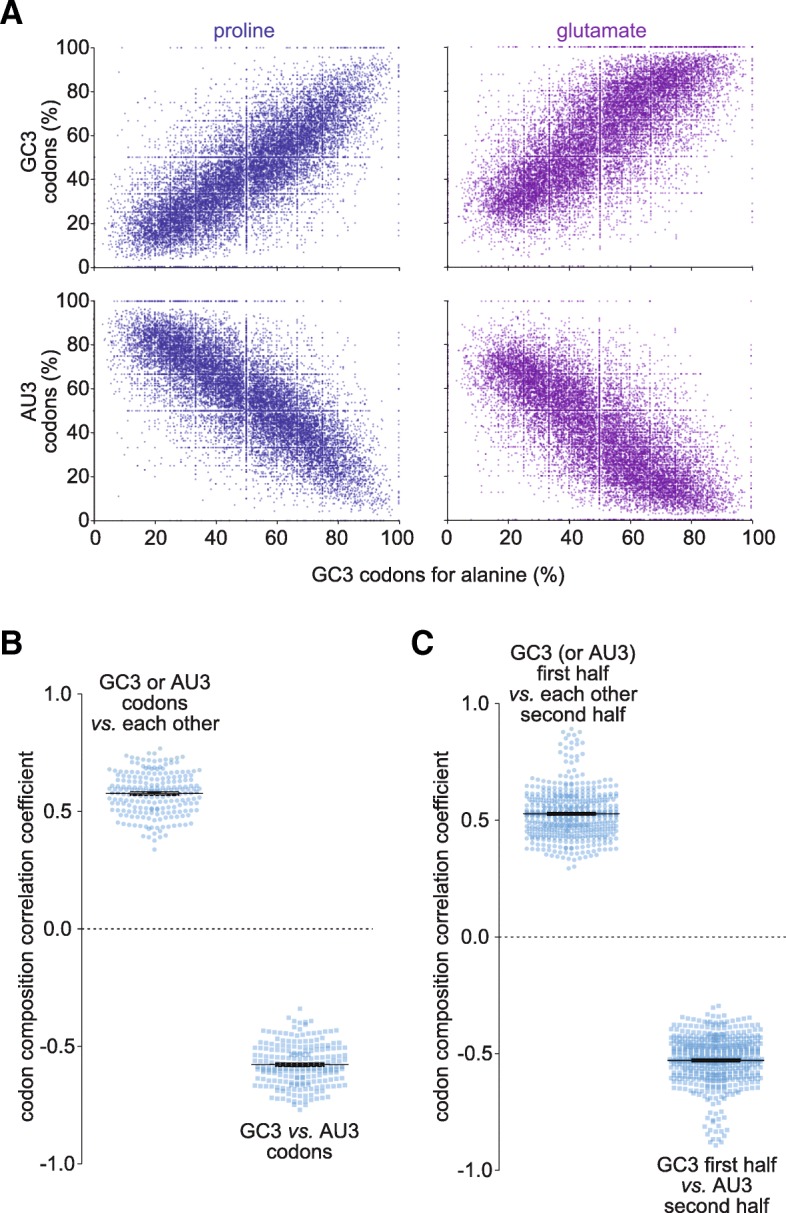
GC3 and AU3 codons are not distributed randomly across coding sequences. **a.** The proportion of the GC3 codons of alanine, calculated as proportion of all codons for alanine in each coding sequence from the human genome plotted against the proportions of either the GC3 or the AU3 codons of proline (blue) or glutamate (purple). Each point represents a different coding sequence (different gene). This implies that if a sequence contains high percentages of GC3 codons for alanine, it will also tend to have high percentages of GC3 codons for either proline or glutamate. **b.** Correlation coefficients resuming all the possible combinations of codons (all amino acids against each other). All GC3 codons within a transcript positively correlate with all other GC3 codons. The same is true for AU3 codons. On the contrary, all GC3 codons anti-correlate with all AU3 codons. **c.** The same analysis as in **b**, performed after splitting the coding sequences in halves, and calculating the correlations between codons from one half, and codons from the other half. The observation from **B** remains valid even in these conditions

One essential aspect to clarify is if the different GC3 and AU3 codons are distributed randomly across the different coding mRNAs, or if their organization follows some principles. For this reason, we analyzed the proportion of all different synonymous GC3 and AU3 codons across all human mRNAs (see two examples in Fig. 2a). This analysis revealed that all GC3 codons correlate positively with all other GC3 codons, and that they anti-correlate with AU3 codons (Fig. 2b). Similarly, all AU3 codons correlate positively among themselves, and anti-correlate with all GC3 ending codons (Fig. 2b). This is true even when these correlations are calculated after halving the transcripts and comparing the codons from one half with the codons from the second half (Fig. 2c).

This implies that the human genome has genes with set fractions of GC3 codons: some transcripts have high amounts of GC3 codons, for all amino acids, throughout their sequence, while on the contrary others have high amounts of AU3 codons, for all amino acids in the sequences.

### The genes rich in AU3 codons (*Group 1, GP1*) are involved in cell growth and proliferation, while the genes rich in GC3 codons (*Group 2, GP2*) are involved in differentiation and specialized cell function

**Fig. 3:**
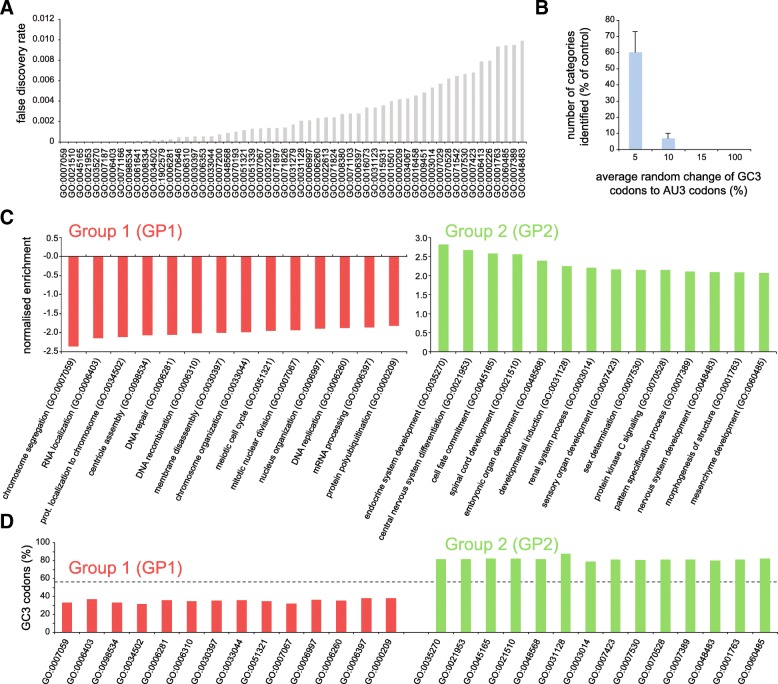
The gene ontology analysis of transcripts ranked by their codon composition (proportion of GC3 or AU3 codons) identifies several categories that can be divided in two subgroups of genes. **a**. Several significant GO categories can be identified on the basis of the percentages of the GC3 codons of human mRNAs. For this analysis we used the WebGestalt 2017 gene set enrichment analysis toolkit [22]. See Methods for details. **b**. Although the gene set enrichment analysis of WebGestalt discards results obtained by chance based on the FDR, we wanted to make sure that the categories identified here truly depend on their GC3 codon percentages. For this purpose, we repeated the analysis after randomly changing the percentage of the GC3 codons for each coding sequence, and we counted the number of gene ontology categories that were identified with a false discovery rate (FDR) below 0.01, in at least 5 different replicates. Changing the average GC3 percentage by as little as 5% already reduced the number of GO classes that were detected. A 15% change completely abolished the detection of GO categories. **c**. Some exemplary GO categories are shown for Group 1 (GP1) and Group 2 (GP2) genes. The two groups of genes are connected with completely different biological processes. GP1 genes are important for cell division and cell cycling, while GP2 genes mediate cell differentiation and functions that arise in specialized organs. The Y-axis indicates the normalized gene enrichment in GC3 codons; high negative values refer to genes containing high levels of AU3 codons. **d**. Difference in codon composition between the Group 1 genes (GP1), and the Group 2 (GP2) genes. The GP1 genes are rich in AU3 codons while GP2 genes are rich in GC3 codons. The segmented line indicates the average percentage of GC3 codons in all coding mRNAs of humans

The fact that GC3 codons tend to be found in the same genes, while avoiding AU3 codons, is far from the chance distribution. In fact, the probability that this comes from a random (chance) event is 1 in approximately 5.1 × 10^19^, raising the possibility that this is under a positive selection and has evolved to satisfy a specific purpose. To test this hypothesis, we investigated how the percentages of GC3 or AU3 codons in human mRNAs relate to the nature of the genes. In other words, we analyzed the cellular processes that rely on genes with high proportions of GC3 or AU3 codons. Using the WebGestalt 2017 gene set enrichment analysis toolkit [22], we performed a gene set enrichment analysis of transcripts ordered by their GC3 content (see Methods for details). This allowed us to analyze the transcripts ranked for their GC3 content, and to identify the gene ontology (GO) “biological process” categories that are significantly enriched. In simple terms, this reveals the biological processes that correlate with either high or low GC3 content at the mRNA level. A summary of the results is presented in Fig. 3, and is detailed in Additional file 1.

Briefly, more than 60 GOs are identified with a significant false discovery rate (FDR, calculated with the BH approach, as detailed in the Methods) below 0.01, based on the ranked percentage of GC3 (Fig. 3a and Additional file 1). These include several well-known GOs such as “chromosome organization” (GO:0033044), “DNA replication” (GO:0006260) and “cell fate commitment” (GO:0045165).

In principle, if the genes with different codon compositions would be randomly distributed, no GO classes would be ever detected in this analysis. To exclude that our findings are dictated by chance, we repeated the same analysis after randomly changing the percentage of the GC3 codons for an increasingly higher number of coding sequences (Fig. 3b). Changing this value by as little as 5% reduces by about half the number of GO classes that are detected. A 15% change completely abolished the detection of significantly different GO classes. An additional analysis on the nature of the genes that were linked to these GO classes also excluded the possibility that specific protein families with very high percentages of GC3 or AU3 codons would bias our results. In fact, we found that each of the GOs detected was composed of genes from a large variety of families, and thus the influence of individual protein families is entirely negligible.

The enriched GOs that we have identified could be subdivided in two groups: those that are composed by genes significantly de-enriched in GC3 codons (note the negative enrichment values in Fig. 3c, left panel) and those that are composed by genes significantly enriched in GC3 codons (positive enrichment values, right panel). The first group, which we will refer to as Group 1 (GP1), contains genes that are associated to significantly enriched GOs and that have sequences rich in AU3 codons (approximately 600 such genes are shown in Additional file 2; typically ~ 40% more AU3 codons than expected by chance). Vice versa, the second group, which we will refer to as Group 2 (GP2), contains genes associated to significantly enriched GOs that have sequences that are substantially enriched in GC3 codons (approximately 650 genes in Additional file 2; see also Fig. 3d). As expected, the relative synonymous codon usage (RSCU), a common measure of codon bias [23], confirmed that GP1 transcripts prefer AU3 codons, while GP2 have a preference for GC3 codons (see for details Additional file 3).

By a closer inspection of the GOs within the two groups, it became clear that the GP1 and GP2 groups were non-overlapping, and were to some extent functionally opposed to each other (Fig. 3c). Briefly, GP1 genes are connected with processes that are important for cell division and cell cycling, while GP2 genes mediate cell differentiation and specialized functions that arise in different organs (such as renal or nervous system development). These findings are similar to what has been reported by Gingold and collaborators [16], who analyzed the tRNA pools and mRNA levels of cells that were undergoing either differentiation or proliferation programs. Their analysis revealed that 82 genes induced during differentiation and 92 genes induced during cell division have a different and opposite codon usage, and that the tRNA pools in these transitions have anticodons that often match these two usages [16]. Although the logical approaches that we and Gingold et al. followed are completely different, some of the GO categories identified correspond, indicating that GC3 content is an important indicator of a switch in cellular programs.

**Fig. 4:**
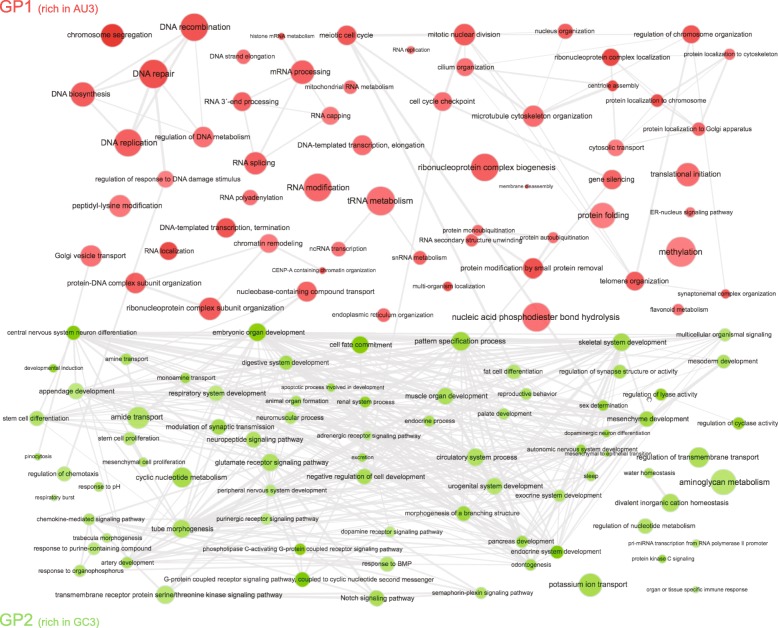
Node-graph visualization of the GO terms describing GP1 and GP2 genes, as determined by the REVIGO algorithm [24]. Larger dots represent more relevant GO terms, characterized by relative enrichment. Note that GP1 and GP2 gene ontology categories are linked within each of the two groups by a higher number of interconnections than across the two groups. This suggests that within GP1 and GP2 there are similarities. Also note that, as detailed in the main test, upon randomization of the genes assigned to the two groups, there is a strong (~ 26 fold) increase of number of interconnections between the two groups, implying that the separation of the two groups of genes is not stochastic. For a list of these categories, see Additional file 1

To investigate this impression further, we visualized all the different GO categories in a node-graph, using the REVIGO algorithm [24], as shown in Fig. 4. Most of the connections were concentrated within each group, and very few connections linked GP1 to GP2. There were on average 0.25 connections for each GP1 term to a GP2 term. To estimate whether the separation of the GP1 and GP2 genes is a stochastic process, we randomized the assignation of the identified GOs to either of the two groups, and we counted the number of interconnections between the newly defined nodes. After randomization there is a ~ 26-fold increase in the interconnection between the two groups (from 0.25 to 6.48 connections between each GP1 node and GP2 nodes). This implies that the separation of the two groups of genes is not stochastic, further reinforcing the idea that AU3 vs. GC3 content is an important discriminator for cellular and tissue function.

We thus conclude that the first set of genes, rich in AU3 codons, the “GP1 genes”, promote cell division, growth and proliferation, while the second set, the “GP2 genes”, and which are rich in GC3 codons, promote completely different processes such as terminal cell differentiation and specialized cell function. Moreover, the division of GP1 and GP2 classes of genes and the underlying GO processes is not stochastic, and the interconnections between the biological processes suggests a rational organization.

As observed in Fig. 1b, some organisms do not show a particularly wide range in the distribution of GC3 codons (as for example yeast). From this perspective, it was interesting to ask whether the GP1 and the GP2 genes from various organisms also have different codon compositions (Additional file 4). The GP1 and GP2 genes are most different in vertebrates, in particular in human and other placental mammals, while other organisms show a more limited difference, as for zebrafish and *Drosophila*, or no difference at all, as for *C. elegans*, implying that this phenomenon is particularly relevant in larger mammals, and may be rooted in vertebrate evolution.

### The genes rich in AU3 codons (*GP1*) are activated in cancer, paralleled by a change in the employment of AU3 and CG3 codons

The opposing nature of the AU3-rich and GC3-rich genes makes it probable that the usage of AU3 and GC3 codons changes in different physiological conditions. Differentiated cells should normally favor GC3 codons, and produce larger mRNA amounts from genes containing such codons [16]. In contrast, proliferating cells in diseases, such as cancer, should employ the GP1 genes, and should therefore preferentially use AU3 codons. To test whether this change takes place during pathophysiological alterations, we performed the following analysis. For each codon we verified whether it is employed in the most abundant transcripts, or in the lower abundance ones. To obtain a precise measure for the codon employment, we calculated for each codon the Pearson correlation coefficient between two vectors: 1) the % of the particular codon in the composition of each transcript (for example, the AAA codon, which encodes lysine, makes up between 0 and 18% of human transcripts, averaging at ~ 2.5%), and 2) the abundance of each transcript. We termed this *codon employment coefficient* (CEC; see Material and Methods for details). In simple terms, this measure reflects how much a codon correlates to the mRNA abundances in a specific dataset. As an example, if a codon makes up a high percentage of the composition of the most abundant mRNAs, its CEC will be high, while if it is used more often in the least abundant mRNA its CEC will be low (negative). Inherently these data demonstrate that a subset of codons was preferentially employed in abundant transcripts (those with the most positive CEC values), while other codons were preferentially found in low-abundance transcripts (those with the most negative CEC values). An analogous concept, termed “codonome”, has been also introduced by Piovesan and collaborators, for which the authors have developed a free software to perform similar calculations [25].

**Fig. 5:**
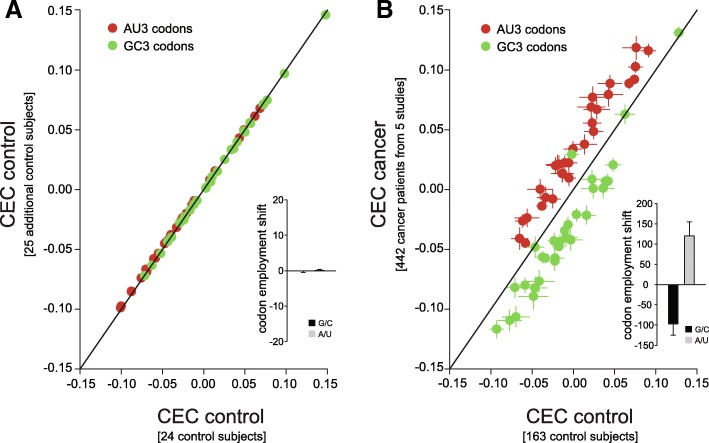
Confirming the existence of the codon employment shift from the available mRNA abundances, and by modeling. **a.** Schematic representation of the two scenarios. On the left, 1st scenario: the shift in codon employment is causal in nature, and drives changes in the gene expression. mRNAs richer in AU3 codons (lighter red color) get more expressed, irrespective of which gene group they belong to. In other words, the shift drives the expression of all transcripts in proportion to their AU3 codon %. On the right, 2nd scenario the shift in codon employment is an effect of the differential expression of GP1 genes. In this scenario GP1 genes are favored, independent of their respective AU3 codon %. **b.** Measured and modeled codon employment shift, analyzed over all genes. When measured across all mRNAs, the codon employment shifts towards the AU3 codons. A model following the second scenario also reproduces the codon employment shift measured in cancer. **c.** We measured (left) or modeled (right) the codon employment shift only in GP1 genes, taken in isolation. The “effect” model no longer reproduces the data. **d.** As in **c** but for all the other genes (not part of GP1). The 2nd scenario is again unable to reproduce the results, which implies that the codon employment shift is probably causal in nature. The codon employment shift is expressed as average CEC change, in % of the initial CEC values

We first calculated the CECs for 49 control subjects from a large cancer study [26]. We than plotted the results after dividing the patients in two groups. As expected, the data from the two groups are very similar, and overlap on the identity line (Fig. 5a). When a similar analysis is performed between cancer patients and control subjects (from several studies, as detailed in the figure legend), a clear shift in the CEC values for most codons is visible. The CEC values for all of the AU3 codons move above the identity line (Fig. 5b), indicating that AU3 codons are indeed preferred in tumor cells, while the opposite takes place for most of the GC3 codons.

### The shift in codon employment appears to drive the transcript abundance changes, rather than being a consequence of these changes

**Fig. 6:**
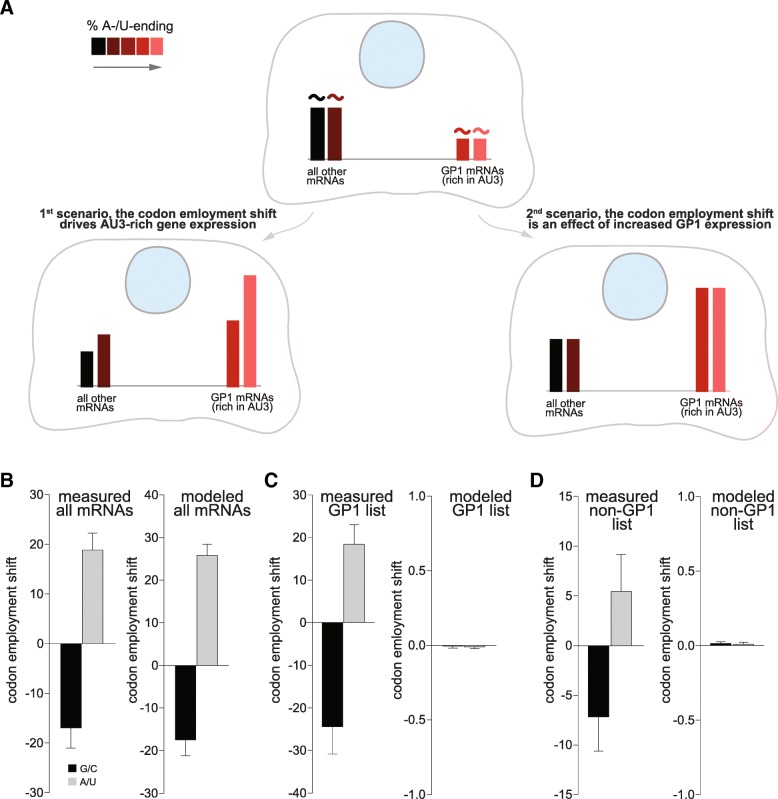
Codon usage shift as a hallmark of human pathology. **a-b.** The Pearson correlation coefficients between the codon percentages in the transcript composition and the respective transcript amounts (CECs) in healthy subjects and in cancer patients **a.** The graph indicates the means ± SEM for the codon coefficients for the mRNA amounts in control subjects. The SEMs are here often smaller than the symbol sizes. Data points show the averages of 24 (X-axis) or 25 (Y-axis) controls subjects analyzed. The black line is the identity line, not a fit to the data, to illustrate the fact that most codons are aligned on the identity line. The inset represents the codon employment shift, expressed as the average percentage change of the CEC. For a detailed description of the CEC, please refer to Materials and Methods. The data analyzed for this panel has been previously published [26]. **b.** Similar graph comparing healthy subjects with cancer patients. In cancer there is a clear shift in codon employment in the A−/U-direction. All AU3 codons are above the identity line, while the GC3 codons are, correspondingly, below the identity line. The inset summarizes this effect as the average codon employment shift (expressed in % of the control CECs, over the different AU3 or GC3 synonymous codons). The data used for this panel is derived from several published cancer studies [27–34]. The error bars indicate the variation between independent studies

One important aspect when considering this codon employment shift is to determine how it might arise. At least two different scenarios can be envisaged. First, a direct scenario: the codon employment shift is *causal in nature*, and, for example, in cancer it drives the production of mRNAs that contain more AU3 codons. It therefore favors the increase of mRNAs from the GP1 genes (as represented on the lower left side of Fig. 6a), and also the increase of mRNAs from other genes that may be rich in AU3 codons. The more a transcript is rich in AU3 codons, the more it will be increased. On average, GP1 genes will be more favored than GP2 genes, since they have far more AU3 codons. At the same time, these effects should also have a “graded magnitude” within the two individual groups. Thus, the “highly AU3-rich” transcripts in GP1 will be increased more than the “less AU3-rich” transcripts in GP1. Moreover, all transcripts from GP2 that contain sizeable levels of AU3 codons will also be favored – they will increase more than the GP2 transcripts that contain virtually no AU3 codons. It is important to notice that if one is to selectively analyze AU3-rich transcripts, there is no a priori expectation that their abundances correlate to the AU3 levels. This means that if they are just expressed independent of each other, there is no particular reason for which very AU3-rich transcripts should be more abundant than moderately AU3-rich transcripts. Conversely, if we analyze only AU3-poor transcripts (GC3-rich ones), there is no reason for very AU3-poor transcripts to have lower abundances than moderately AU3-poor ones. If, however, the codon employment shift is causal in nature, and the organism has somehow preferentially stabilized the mRNAs in the “AU3 direction”, then all transcripts are affected. Very AU3-rich transcripts will become more abundant than moderately AU3-rich constructs, and very AU3-poor transcripts will become less abundant than moderately AU3-poor transcripts.

Second, an alternative scenario: the apparent change in codon employment is just an *effect* of the specific increase in the GP1 gene expression. We have shown that several GP1 genes are connected to cell proliferation, cell division and cell cycling, all processes that are substantially over-represented during cancer transformation. In this scenario, the GP1 mRNA expression is increased by other causes, such as specific regulatory mechanisms that recruit transcription factors that promote cancer progression. This would push the overall codon employment in cancer cells toward the AU3 codon direction, simply because large amounts of A−/U-containing mRNAs (from the GP1 genes) would be now abundant in these cells. Note that in this scenario the levels of a particular transcript are not directly linked to its AU3 content. The “highly AU3-rich” transcripts in GP1 are not necessarily increased more than the “less AU3-rich” ones. Similarly, the AU3-richer transcripts in GP2 are not more abundant than those that contain virtually no AU3 codons.

To differentiate between these two scenarios (depicted in Fig. 6a) we can rely directly on the available human pathology datasets. In the first, causal scenario, the shift in codon employment should be measurable on any selected set of genes: all genes, GP1 genes alone, GP2 genes alone, or any other set, and be only dependent on the relative AU3 percentages. In the second scenario, the shift in codon employment can only be measured when considering all genes, but is not visible when analyzing for example GP1 genes in isolation. We used the measured results from Fig. 5 for this analysis resumed in Fig. 6b-d. In detail, we measured (left) or modeled (right) the codon employment shift for all mRNAs or only in GP1 or in non-GP1 genes, taken in isolation. While the model works for all the mRNAs, it no longer reproduces the data when considering GP1 genes in isolation. In simple terms this indicates that even within the GP1 genes, those that have higher AU3 levels are more increased than those with lower AU3 levels (Fig. 6c). The codon employment shift is even noticeable for genes that are not part of the GP1 (Fig. 6d). Overall, these results indicate that the first scenario is the valid one.

We therefore conclude that cancer induces a global shift in the codon employment that can be highlighted by calculating the CEC. While in normal tissue the GC3 codons are preferred, in cancer the opposite takes place, and the AU3 codons are preferred. As a result, the cohort of genes that we termed GP1 (involved in cell proliferation ang growth) is favored by the global codon employment shift that is occurring in this pathology.

### The CEC analysis is robust and can be applied to any mRNA abundance dataset

The previous section suggests that the expression of individual genes is modulated by the shift in the CECs. A simple assumption would be that if the composition of a transcript fits more closely to the new CECs, its expression would be favored upon the shift from the old (normal) to the new (pathological) CECs. For example, the coding sequence of the gene encoding the neuronal exocytosis protein synaptobrevin-2 (VAMP2), has 72.65% GC3 codons, and just 26.5% AU3 codons. This sequence “fits” better to the codon employment in normal tissue (which favors GC3 codons) than to the codon employment in cancer tissue (which favors AU3 codons). In principle, this gene should therefore be more strongly expressed in normal tissue than in cancer tissue. This was indeed the case in the cancer studies we analyzed.

**Fig. 7:**
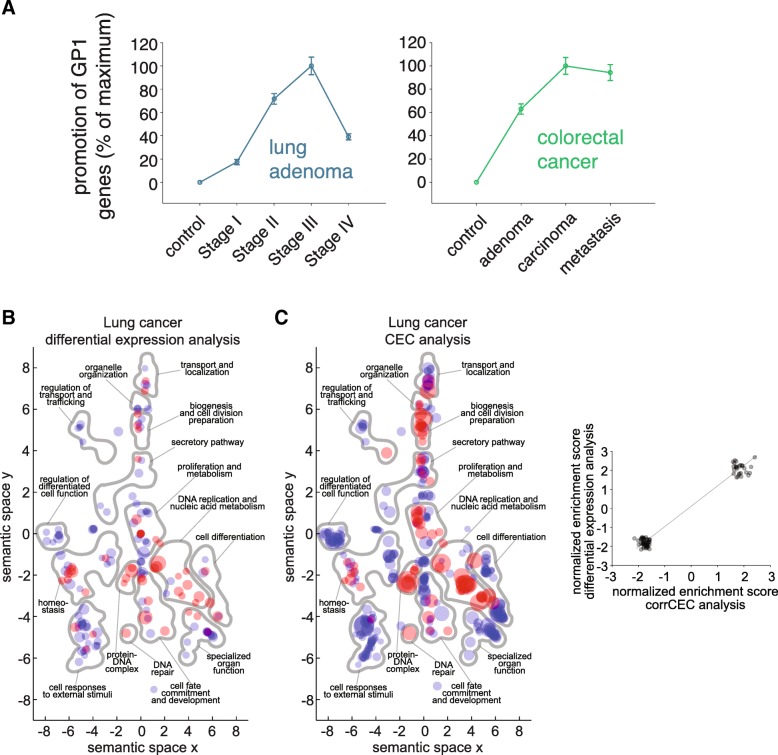
Codon employment shift as a commonality of pathological changes. **a.** The behavior of GP1 genes in different stages of cancer. The graphs indicate that GP1 genes are associated to early cancer stages, and that they become even more strongly favored as the cancer progresses, although this tails off in the last metastasis stages, when the cancer tissue attains its maximal development. The symbols show the average differences in CEC in cancer versus controls for the GP1 genes, in several stages of non-small cell lung cancer [27] or colorectal cancer [28], normalized to the maximal change observed in the respective study. **b-c.** Gene ontology semantic signatures of differential expression **b** and CorrCEC analysis **c**. Red spots indicate significantly enriched “biological process” GO categories, while blue spots indicate de-enriched categories. The symbol size is proportional to the magnitude of the change. The significant enrichment here refers to GO categories containing significant amounts of genes whose sequence codon compositions correlate better to the codon employment observed in disease; de-enrichment refers to GO categories containing genes whose codon compositions anti-correlate to the codon employment observed in disease. We plotted the significantly enriched GO categories using their X- and Y- semantic coordinates to represent their semantic signature (Additional file 6; [24]). For more detailed information about these graphs see Additional file 5 and the examples detailed in Additional files 8, 9, 10, 11, 12, 13, 14, 15, 16 and 17. The inset on the right represents a scatter plot of the enrichment scores for the two analyses, and confirms the broad overlap between the codon employment-predicted expression and the actual mRNA expression in cancer tissue

To investigate this over all transcripts, rather than just for synaptobrevin-2, we calculated the correlation of the transcript composition (expressed in the form of 61 codon percentages) to the CECs before and after disease onset. We termed this “correlation of transcript composition to codon employment”, or CorrCEC. For this measure, the codon composition of each transcript, in %, was again determined (consisting of 61 codon percentages). The codon composition was correlated to the codon CECs in controls and in disease samples, for every single transcript. In simple terms, this verifies whether the composition of the respective transcript more closely correlates to the preferred codon employment in disease or in the control situation (where negative values indicate a correlation to the preferred codon employment in the control situation, while positive values show a correlation to the disease situation). We applied this analysis to the cancer study mentioned above, and we found, unsurprisingly, that the GO categories of GP1 were favored, while GP2 genes were disfavored. Importantly, this analysis determined that the codon employment shift was not a binary process. The shift could be stronger, or less strong, according to the type of cancer and the stage, as indicated in Fig. 7a.

The actual genes and GOs that were determined as favored overlapped broadly with the genes for which the mRNA expression was increased, as determined by direct mRNA abundance measurements (Fig. 7b-c, Additional files 5 and 6). However, the CorrCEC analysis detected more gene groups than the conventional differential expression, with higher sensitivity (see also the Additional files 8, 9, 10, 11, 12, 13, 14, 15, 16, 17 and 18).

The difference in sensitivity can be due to two possibilities: 1) the effects of the codon employment shift are counteracted by other cellular mechanisms, and thus are not detected by expression analyses; 2) the experimental mRNA readings are too noisy (variable) to reveal some of the changes in expression, which are nonetheless predicted by CorrCEC. This analysis relies on the measurement of codon correlations to mRNA abundance, across thousands of genes, and therefore has an extremely limited noise from patient to patient (Fig. 5a). As discussed later, this analysis is little affected by randomly changing the mRNA readings for the individual genes by a factor of up to 10-fold (see for details Fig. 9b, below). The direct mRNA measurements are far less noise-resistant, since each gene is read and treated independently. Therefore, it is not surprising that CorrCEC can detect more effects that would be missing, due to noise, in the measured data set.

### The codon employment shift is a common signature for a multitude of other human diseases, and can be used to generate diagnostic procedures

**Fig. 8:**
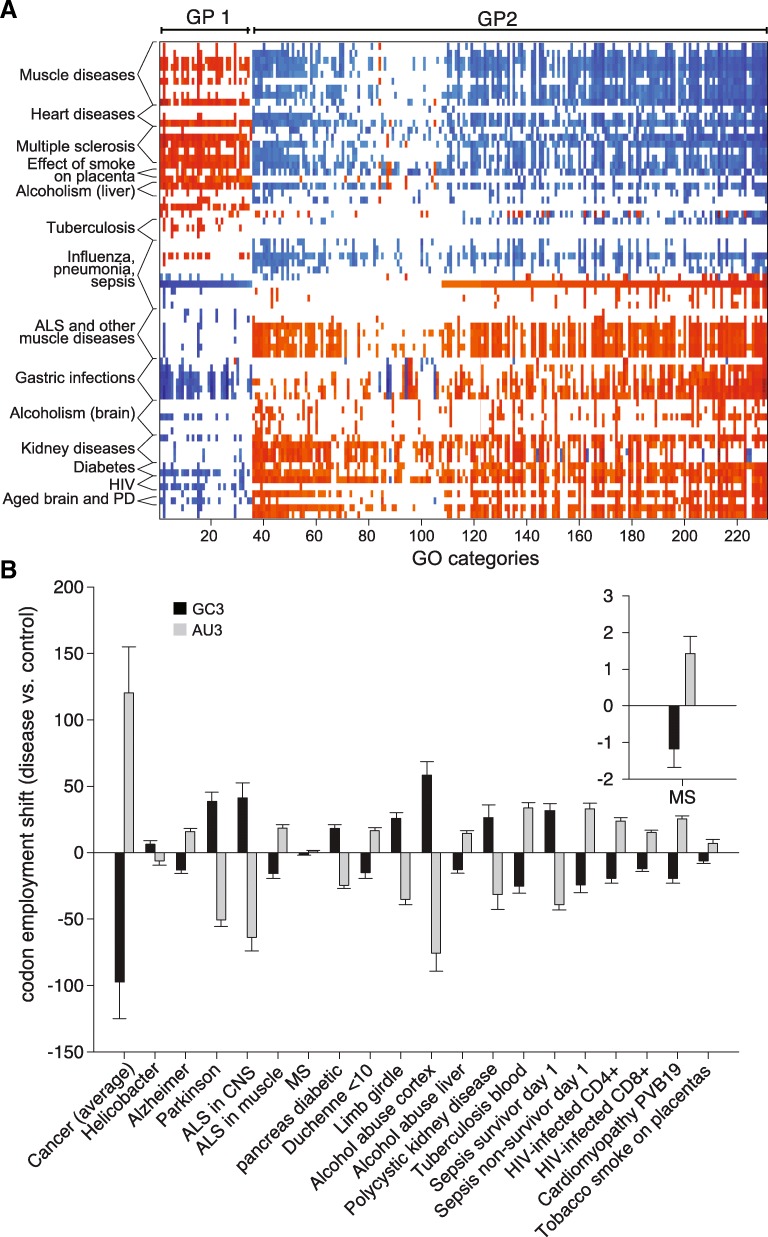
Applying the analysis of the codon employment shift to several diseases. **a.** Graphic depiction of the favored or disfavored GO categories in multiple diseases (see Additional file 7 for detailed references of the datasets taken into consideration). The Y- axis indicates different diseases, while the X-axis indicates different GO categories that were significantly correlated to the employment shift (red) or anti-correlated (blue) in at least 10 different diseases. The different colors indicate the normalized enrichment score, calculated as in Fig. 7. The first 35 GO groups belong to the GP1 genes. Several more detailed examples are included in Additional files 8, 9, 10, 11, 12, 13, 14, 15, 16 and 17. **b.** Codon employment shift in the different diseases, calculated as for the insets of Fig. 6A-B. The inset highlights the smallest change measured, in multiple sclerosis (MS). ALS = amyotrophic lateral sclerosis. Cardiomyopathy PVB19 = cardiac inflammation and damage following parvovirus infection. We only employed in this figure studies that also provided sufficient numbers of control (non-disease) patients

We followed upon the previous cancer observations by applying the same analysis for many diseases. We present multiple cases in Fig. 8, based on published data from several studies (see Additional file 7; the studies used in this figure belong to the first 70 lines of the table). Each disease induced a slightly different codon employment shift, which we measured across all genes, using CorrCEC. Then, using the same GO analysis presented for Fig. 7b, we determined the GO protein categories that would be favored by the codon employment shift, and those that would be disfavored. This provided a gene ontology signature that resembles a barcode for each disease (Fig. 8a).

To summarize these observations, we also calculated the codon employment shift between the different diseases and the controls, in the same fashion we did for the insets of Fig. 5. The diseases taken into consideration shift the codon employment in different directions, by different magnitudes (Fig. 8b). As an example, multiple sclerosis (MS) has a small but significant shift for the codon employment in the AU3 codon direction (inset in Fig. 8b), while Parkinson’s disease and amyotrophic lateral sclerosis (ALS) in the gray matter correlate to a codon employment shift towards the GC3 codon direction, probably in the effort of promoting GP2 genes, and thereby inducing the differentiation of neuronal precursors (for a more detailed discussion about the codon employment shift in different diseases see Additional files 8, 9, 10, 11, 12, 13, 14, 15, 16 and 17). The shift in codon employment observed in pathology might reflect in some cases the use of the codon employment as a pathological mechanism (as observed in cancer), and in other cases the physiological response put in place by the organism to fight the pathological changes. A prominent example is the shift in the proliferation (AU3) direction for young (< 10 years) Duchenne dystrophy patients, in which the myocytes have to constantly multiply to replace damaged muscle fibers. This is a mechanism that compensates for muscle degeneration at these ages (see Additional file 12).

**Fig. 9:**
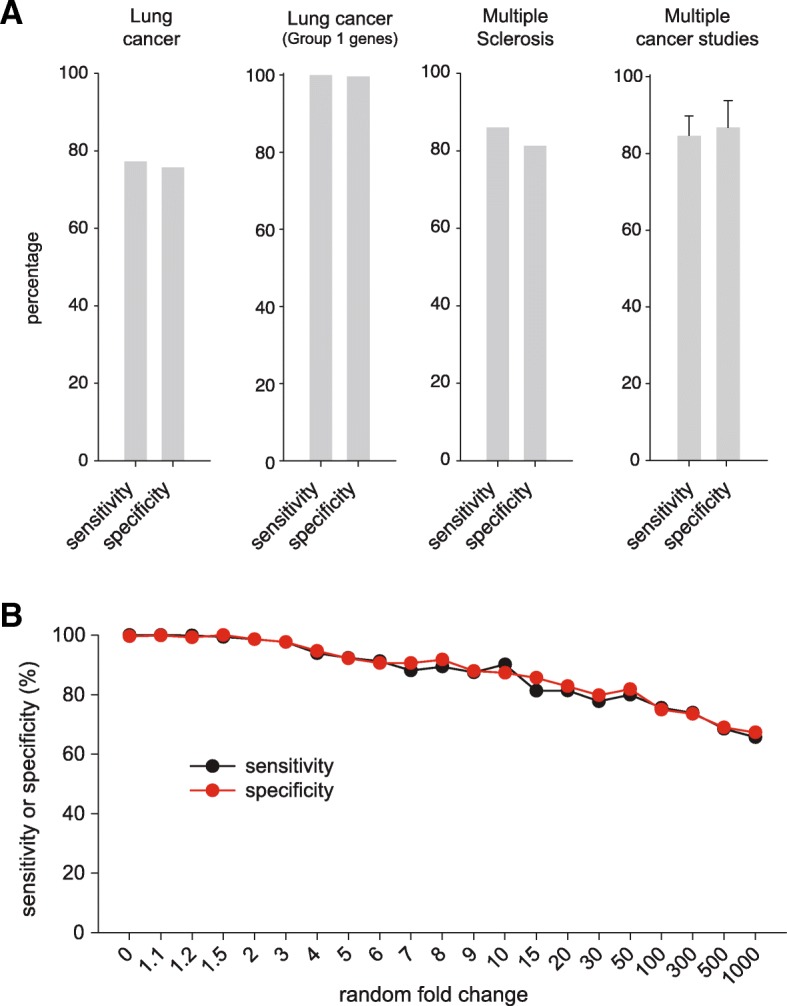
The sensitivity and specificity of diagnostics based on the codon employment shift. **a.** We used transcriptomics data from the following studies: a lung cancer study for the first two graphs [27]; a multiple sclerosis study for the third graph [35]; data for the multiple cancer studies for the fourth graph [27–30]. Please refer to the main text for more information on how the diagnostic approach was designed. The bars show the sensitivity and specificity (as defined in medical diagnosis where the sensitivity is the ability of a test to properly recognize patients with the disease and the specificity is the ability of the test to properly recognize those free from the disease). **b.** The sensitivity (black) and the specificity (red) of the lung cancer diagnosis under conditions of randomized mRNA abundances. The mRNA abundance values in patients and controls were randomly changed, up or down, by a fold factor indicated on the X-axis. The same diagnostic analysis as in Fig. 9a then followed. Changing randomly all measurements by up to 10-fold leaves the precision of the diagnostics within the clinically accepted limits (85%)

To test whether diagnostics could be based on these observations, we returned to a comprehensive cancer study, which was already used in Figs. 5 and 7 [27]. We separated the cancer patients and the controls in training groups (80% of the data) and testing groups (20% of the data), and then performed the CorrCEC analysis for all patients in the training groups. We used the training groups to generate a CorrCEC cutoff that separated the cancer patients from the controls, and we measured the accuracy of this cutoff in the testing groups. The procedure was repeated 1000 times, randomizing the training and testing groups. The results are shown in Fig. 9a. We measured the sensitivity of the diagnostic (defined as the percentage of cancer patients that were correctly identified) and also its specificity (defined as the percentage of controls that were correctly identified). Applying CorrCEC across all genes, both of these values were around 75–80%. However, a selective analysis of the GP1 genes resulted in a far higher sensitivity and specificity – over 99.5%.

We then repeated this second analysis, based on CorrCEC applied on GP1 genes, for the disease that provided the smallest codon employment shift in Fig. 8, multiple sclerosis, which is also a disease that has been notoriously difficult to diagnose. We obtained sensitivity and specificity values of ~ 85%, which is the typical acceptable medical cutoff for diagnostics.

We also confirmed the reliability of our CorrCEC analysis across multiple studies. Expression analysis is known to fail when applied across multiple studies. To this purpose, we applied CorrCEC analysis to 5 types of cancer (lung cancer, colorectal adenoma, carcinoma and metastases, and brain tumors), from four independent studies. In detail, we trained a CorrCEC cutoff (again on GP1 genes) on the basis of the difference between normal human tissues [36] and cancer cells [37], and then applied this cutoff to different studies [27–30]. The resulting sensitivity and specificity values averaged ~ 84–87% (Fig. 9a, rightmost panel).

Finally, we set out to test whether these diagnostics require extremely accurate mRNA data, obtained through expensive high-quality analyses, or whether substantially cheaper, but more inaccurate readings, such as those obtained with Oxford Nanopore devices or “Human amplicome” next generation sequencing type of measurements with very low coverage (< 0.3 M reads), would suffice. Our analysis only requires an understanding of the overall shift in codon employment, and therefore should be little affected by the noise of inaccurate recordings. This was indeed the case. Changing all mRNA abundance measurements randomly by up to 10-fold still allowed the diagnostic to remain within the 85% sensitivity and specificity limit required for clinical studies (Fig. 9b).

## Discussion

Here, we used a systems medicine approach to study in detail mRNA transcript composition in terms of the third (wobble) nucleotide. We combined the analysis of wobble codon usage in humans and other organisms to the study of 40 human pathologies, for which extensive transcriptional datasets have been published.

A first result is that a group of genes significantly enriched in AU3 (that we named GP1) are connected to proliferative processes such as cell division and DNA metabolism. On the contrary, genes enriched in GC3 (named group GP2) correlate with a cellular differentiation program, which corresponds to terminal specification into organs and tissues. These finding are only in part new, since it was previously shown by Gingold and collaborators that the GC3 usage in a selected group of differentiation or cell division genes is polarized [16]. Our analysis, which revealed many more genes, started from a completely different set of data (the entire transcriptome), and was thus unbiased, since we were not specifically looking into a specific subset of genes. For this reason, the GO categories that we identify, although in part overlapping with the genes previously reported, extend widely the number of processes that are linked to wobble nucleotides.

This unexpected genome architecture is unlikely to have been reached through random processes, as noted in Results. It has been proposed that it arises as a side effect of a non-adaptive GC-biased gene conversion, which happens during meiotic recombination, and which would maintain the AU3-rich bias of proliferation genes, but without any functional benefit for either proliferation or differentiation genes and processes [2]. In contrast, our results suggest that the GC3/AU3 bias has a role in promoting the expression of specific genes in particular pathophysiological conditions (Fig. 6). For this reason, while we agree that non-adaptive GC-biased gene conversion might concur for the generation of these differences, we cannot exclude that additional selective pressures have acted to optimize the translation of particular sets of genes in distinct cellular states. For example, in cancer it has been observed that specific tRNA genes are causally linked with pathology, and that their modulation interferes with tumorigenesis [17], which is fully in line with the preferential employment of particular codons in cancer. Finally, multiple observations from the literature suggest that the codon usage bias is profoundly linked with numerous other regulatory steps, including maintenance of protein homeostasis and stability. Thus, it is very unlikely that a profound codon usage difference (such as that between GP1 and GP2) has no functional consequences. At the same time, this implies that a variety of multidisciplinary approaches may be necessary in order to understand this issue thoroughly.

One obvious fallacy of our work is that it does not provide a molecular mechanism for the shift in the codon employment in health and disease. As indicated in Fig. 6, it is apparent that the shift is not simply due to the stronger expression of particular sets of genes. In contrast, the more the composition of a transcript aligned with the codon employment, the more that transcript was expressed, in line with the hypothesis that the codon employment shift is a cause of the change in gene expression profiles (albeit probably not the only cause). We cannot offer here a mechanism for how this shift would cause differential gene expression. A multitude of mechanisms could be involved, ranging from changes in the stability of transcripts rich in particular codons [38, 39] to changes at the chromatin level [40]. Testing the various mechanisms would imply a substantial effort, which is beyond the purpose of this work.

## Conclusions

Our approach provides a useful workflow for the molecular characterization of several human pathological states, and enables a simple and reliable analysis of mRNA datasets for diagnostic purposes. The CorrCEC analysis, which is based on measuring the codon coefficients to the mRNA abundance, can be used to discriminate disease patients from controls, and, since it is based on a global change over thousands of genes, it is far more noise-resistant than other approaches, and may perhaps be extended to low-quality patient data. For this reason, this analysis would enable approaches based on inexpensive mRNA readings, which would be within the budget of most clinics.

However, this analysis should not be applied separated from other clinical investigations. The codon employment shift alone may not be sufficient to indicate the nature of the disease. Several disease profiles appear similar (Fig. 8), and it is possible that the diseases cannot be differentiated by such profiles alone (something that should be tested in multi-disease studies with even larger patient numbers). To correct for this, it will be necessary to follow the appropriate clinical practice, and to compare the appropriate samples between healthy (control) and disease-suspected patients. This procedure is, for example, routinely employed during biopsy investigations, and should be sufficient to differentiate between most of the diseases. To exemplify this further, although the codon employment shifts may be similar for lung cancer cells and Duchenne dystrophy cells, the two diseases are immediately separated in clinical practice, by investigating either lung nodules, as customary when lung cancer is suspected, or muscle fibers, as for suspected Duchenne dystrophy.

An additional advantage of CorrCEC is that it reveals the genes that may be especially promoted during disease, independent from the transcriptome measurements for the particular genes. Genes that are transcribed at low levels, and whose expression changes are indistinguishable from noise, but which may still be important in disease, might be identified in this manner.

Finally, our work offers new arguments in the current dispute over the suitability of animal models for human disease research. Since the differences in GC3 codon employment are very limited in mouse, and virtually absent in zebrafish and invertebrates, we suggest that human samples, including iPS cells and iPS-derived tissues, could be essential for the correct understanding of some pathologies, including cancer and neurodegenerative diseases.

## Methods

The aim of this study was 1) To provide a descriptive characterization of AU3 and GC3 usage in vertebrate (mammalian) genomes and 2) Study the possibility that shifts in the employment of synonymous codons can be used for predicting and possibly understand pathological alterations in humans. This study stems from previous observations where we found that codon usage is linked to protein stability [11, 12].

### External datasets

All the human external datasets used in this work are summarized in Additional file 7. All mRNA sequences used for calculating the with and distribution of GC3 were downloaded from Ensembl [41], and the latest updated assembly for each organisms was used.

### Data analysis

All analyses were performed with custom-build MATLAB scripts (MathWorks). All scripts are available from the authors upon reasonable request.

### Calculation of AU3 and GC3, RSCU and correlation calculations

The average content of each transcript, in codon percentages was calculated for all codons, after converting the sequences obtained from Ensembl BioMart into codons (Fig. 1). The correlation between different codons (Fig. 2) was calculated from this dataset, by simply cross-correlating the values for the different codons, across all transcripts. For Fig. 2c, the sequences were split in halves, and this analysis was repeated.

The relative synonymous codon usage (RSCU), a common measure of codon bias, was calculated on the GP1 and GP2 genes summarized in Additional file 2 as previously described [23].

The CEC was calculated as follows. For each codon we determined the % it represents of the composition of each transcript. We then correlated this set of values (consisting of 61 codon percentages) with the abundance of the transcripts. The resulting Pearson’s correlation coefficient represents the CEC. In simple terms, this measure reflects how much a codon correlates to the mRNA abundances in a specific dataset. As an example, if a codon makes up a high percentage of the composition of the most abundant mRNAs, its CEC will be high, while if it is used more often in the least abundant mRNA its CEC will be low (negative).

The “correlation of transcript composition to codon employment”, or CorrCEC (Figs. 7 and 8), was determined as follows. The codon composition of each transcript, in %, was determined (consisting of 61 codon percentages). The codon composition was correlated (for every single transcript) to the codon CECs in control and in disease samples. In simple terms, this verifies whether the composition of the respective transcript more closely correlates to the preferred codon employment in the disease or in the control situation. Negative CorrCEC values indicate a correlation to the preferred codon employment in the control situation, while positive values show a correlation to the disease situation. Overall, the CorrCEC can be used to pinpoint genes whose composition (in terms of codons) mirrors the codon usage in disease or in the control situation.

For the diagnostics approach (Fig. 9), we separated the patients from the studies mentioned in the main text into training groups (80% of the data) and testing groups (20% of the data). We then performed the CEC analysis for all patients in the training groups. We used the training groups to measure the average CEC shift between the cancer patients and the controls, and we obtained a CEC shift cutoff that would separate well between the groups of patients. We then measured the accuracy of this cutoff in the testing groups. The procedure was repeated 1000 times, randomizing the training and testing groups. The values in Fig. 9 report the accuracy determined in the testing groups. We trained the CEC shift cutoff on the basis of the difference between normal human tissues [36] and cancer cells [37], and then applied this cutoff to different cancer studies.

### Gene ontology (GO) categorization analysis and visualization

For the GO enrichment analysis introduced in Fig. 3 we used the WebGestalt 2017 gene set enrichment analysis toolkit [22]. In detail we first identified the transcripts corresponding to all reviewed human proteins form the Uniprot database [42]. This allowed us to avoid the influence from badly GO-annotated transcripts (whose proteins are also not reviewed). This selection included 19′007 transcripts (mRNAs) for which we calculated the GC3 content. We than normalized the GC3 content by the average GC3 content of all these transcripts (58.30) and we calculated the log_2_ ratio for all these transcripts with respect to this average. We then used the id of each transcript and the log_2_ ratio as an input for the Gene Set Enrichment Analysis (GSEA) on WebGestalt looking into the functional database: “geneontology>Biological_Process_noRedundant”.

This analysis revealed more than 60 GO identities whose false discovery rate (FDR) was significant (< 0.01), as detailed in Additional file 1. The FDR was obtained in the GSEA analysis through the default mode (BH) and thus calculated by the software with the Benjamini and Hochberg approach [43]". For the node-graph visualization of GO categories we relied on the REVIGO algorithm [24] and used the standard visualization of all the GO categories identified as significant in our WebGestalt analysis to map them on the x and y semantic space. The GOs distributed on the coordinates used in the representation of the semantic space from Additional file 5 and the final values of the x and y coordinates for each of the mapped GOs are available in Additional file 6.

## Additional files

Additional file 1: **Table S1**. Gene ontology categories significantly enriched in GP1 and GP2 transcript groups. (XLSX 16 kb)

Additional file 2: **Table S2**. List of human transcripts significantly enriched in either AU3 (GP1) or GC3 (GP2) codons in the GO analysis (see Methods for details). (XLSX 111 kb)

Additional file 3: **Figure S1**. Relative synonymous codon usage (RSCU) calculated in GP1 transcripts (**A**) and in GP2 transcripts (**B**) as previously described [23]. The analysis confirms that GP1 transcripts are enriched for AU3 codons, while GP2 preferentially use GC3 codons. (EPS 1306 kb)

Additional file 4: **Figure S2**. Contents of GC3 codons in GP1 or GP2 genes for several organisms. GP1 genes are rich in AU3 codons while GP2 are rich in GC3 codons with few small exceptions. Differences are more pronounced in the larger placental mammals. (EPS 851 kb)

Additional file 5: **Figure S3**. Semantic signatures for gene ontology (GO) molecular processes, representing the codon employment shift for GP1 and GP2 genes. The most prominent semantic areas have been grouped. **A.** Representation of a situation where GP1 genes are favored and GP2 are disfavored by the codon employment, as in cancer. **B.** Representation of the opposite situation where GP1 genes are disfavored and GP2 are favored by the codon employment, as in Parkinson’s disease. (EPS 2302 kb)

Additional file 6: **Table S3**. Semantic coordinates of the GOs represented in the graphic depiction of the favored or disfavored GO categories in multiple diseases (Detailed list of coordinates). (XLSX 41 kb)

Additional file 7: **Table S4**. Published data from multiple studies analyzed in this work (References for the studies used in this work). (XLSX 16 kb)

Additional file 8: **Figure S4**. Lung cancer and Helicobacter−positive Gastric epithelium. The lung cancer **A.** is accompanied by a strong increase in the Group 1 genes, which is visible in both the measured differential expression of all genes (left) and in the codon employment shift measurement (right). The opposite phenotype is takes place for the gastric epithelium **B.** upon Helicobacter infection, suggesting that the epithelium is driven to differentiation, rather than proliferation (at least at this initial stage of the disease, before induction of malignant development). For an explanation of the figure and of the analysis see Additional file 5. (EPS 3438 kb)

Additional file 9: **Figure S5**. Alzheimer’s disease and Parkinson’s disease. The brain in Alzheimer’s disease **A.** appears to be dominated by the increased expression of GP1 genes, which is in line with the inflammation and microglia proliferation known to take place in this condition. Interestingly, the cases of Parkinson’s disease that we studied **B.** point to the opposite development, which is more in line with an attempt of generating differentiated cells from non-specialized precursors. This latter phenotype is virtually identical to that observed in the aged brain (70–104 years old). (EPS 3280 kb)

Additional file 10: **Figure S6**. Spinal cord gray matter and muscle of ALS patients. In the case of ALS, the gray matter **A.** shows a cell differentiation phenotype, akin to the one observed in the aged brain or in Parkinson’s disease. In contrast, in the muscle **B.** this phenotype is reversed, with the GP1 genes favored, resulting in cell proliferation. This is in line with the muscle cell division and muscle fiber regeneration that is observed in ALS. Panel A. also showcases the power of the CorrCEC analysis, which detects many significantly favored or disfavored gene groups, although the analysis based on differential gene expression alone is not sensitive enough for this. This is most likely due to the fact that a difficult tissue is analyzed in A., cells laser-dissected from human tissue, which provides more error-prone measurements than, for example, the muscle tissue from panel **B**. (EPS 2383 kb)

Additional file 11: **Figure S7**. Multiple sclerosis and Pancreas of diabetic patients. Multiple sclerosis **A.** induces the growth and proliferation (GP1 genes probably favored) of blood immune cells. This phenotype is difficult to detect using differential gene expression, in line with the fact that multiple sclerosis has been very difficult to diagnose in the past. Similarly, diabetes **B.** effects are difficult to detect by differential gene expression, but the codon employment shift reveals that some GP2 genes are strongly favored, suggesting that more cells tend to differentiate in the diabetes pancreas than in the normal pancreas. (EPS 2675 kb)

Additional file 12: **Figure S8**. Duchenne muscular dystrophy and Limb−girdle muscular dystrophy. These two neuromuscular diseases are caused by mutations in different muscle proteins. The Duchenne dystrophy, caused by mutations in the dystrophin gene, does not present a phenotype before the age of 10, since the muscle cells strongly proliferate, and dying muscle fibers are quickly replaced. This is evident in panel **A**, when using CorrCEC. However, almost nothing is picked up by the direct analysis of differential mRNA expression, in line with the fact that the muscles of a Duchenne dystrophy patient under the age of 10 show virtually no phenotype. It is only when we investigate how the cells adjust their codon employment to counteract the disease, and to prevent the phenotype from developing, that significant changes are seen (right panel). The opposite phenotype (increased differentiation of cellular precursors) takes place in limb-girdle muscular dystrophy **B**, shown here from adults in which the cell proliferation is no longer as potent as in **A**. Once more, the differential expression analysis is unable to reveal substantial changes. (EPS 2343 kb)

Additional file 13: **Figure S9**. Brain frontal cortex and liver of alcohol abuse patients. Another example of codon employment shift favoring differentiation or proliferation in different tissues can be found by investigating the effects of alcoholism in the cortex **A** or in the liver **B**. GP1 (proliferation) is favored in the liver, while GP2 (differentiation) is favored in the cortex. A simple interpretation is that the damaging effects of alcohol abuse lead to proliferation and growth of the liver, to improve the detoxification of the organism. This mechanism is not available for the brain, which therefore needs to resort to the same mechanism as in the aged brain, or in Parkinson’s disease: the differentiation of (presumably) precursor cells. (EPS 2666 kb)

Additional file 14: **Figure S10**. Polycystic kidney disease and blood of Tuberculosis patients. Kidney failure in polycystic kidney disease **A.** is associated with cellular growth, resulting in cysts. Remarkably, medium-size cysts seem to be dominated by a stable form of the cells, which are driven to differentiation, and not to further growth. The opposite is seen in the blood of tuberculosis patients **B**, due to the increased activation, and presumably proliferation, of immune cells. (EPS 2730 kb)

Additional file 15: **Figure S11**. Blood from sepsis survivor and non-survivor patients. An interesting difference has been observed when comparing sepsis patients. Survivors **A.** appear to have their immune cells dominated by an activation and/or differentiation phenotype (possibly in response to the sepsis), while those from non-survivors **B.** seem to have already shut off most genes. (EPS 2036 kb)

Additional file 16: **Figure S12**. HIV-infected CD4^+^ and CD8^+^ cells. Both CD4^+^ **A.** and CD8^+^ **B.** cells are induced to proliferate in HIV-infected patients (GP1 is favored). Interestingly, in both cases the simple differential analysis of genes expression only reveals one subset of genes – the ones that are favored (the ones whose expression increases). The sensitivity of gene expression analyses is not sufficient to reveal that large numbers of GP2 genes are disfavored. (EPS 2778 kb)

Additional file 17: **Figure S13**. Cardiomyopathy inflammation and effect of smoking on placentas. Cell proliferation is also triggered by parvovirus infections of the heart, which lead to cardiomyopathy inflammation **A**. This presumably serves as a compensatory mechanism, to replace the damaged cardiomyocytes. A similar effect (GP1 genes enhanced, GP2 genes depressed) is found in the placenta of pregnant heavy smokers **B**. Importantly, the genes responsible for cell differentiation to neuronal phenotypes, and for the specialized organ functions, seem to be disfavored. The potential effects could be severe for brain development, should this effect also take place in the embryo. (EPS 2518 kb)

Additional file 18: Supplementary Text. File detailing the comments of the reviewers and the answers of the authors. (PDF 90 kb)

## Data Availability

All data is publicly available. All analyses were performed with custom-build MATLAB scripts (MathWorks). All scripts are available from the authors upon reasonable request.

## Abbreviations

ALS: Amyotrophic lateral sclerosis
AU3: Codons ending in adenine or uracil (AU3)
CEC: Codon employment coefficient
CorrCEC: Correlation of transcript composition to codon employment
FDR: False discovery rate
GC3: Codons ending in guanine or cytosine
GP1: Group 1 of genes (associated to cell proliferation and rich in AU3)
GP2: Group 2 of genes (associated to cell terminal commitment and rich in GC3)
MS: Multiple Sclerosis
RSCU: Relative synonymous codon usage
