# Supplementary material for: Pathological changes are associated with shifts in the employment of synonymous codons at the transcriptome level
Source: BMC Genomics. 2019 Jul 9;20:566. doi: 10.1186/s12864-019-5921-9 (PMC6617700; doi:10.1186/s12864-019-5921-9)
Supplement: Supplementary file 18 — Supplementary Text. File detailing the comments of the reviewers and the answers of the authors. (PDF 90 kb) [file 12864_2019_5921_MOESM18_ESM.pdf]

Answers to the comments from the Reviewers' reports

Note: the comments are reported in italicized gray text and our answers are typed in black.

Reviewer reports:

Reviewer 1:
The paper of Fornasiero & Rizzoli report a bias usage in GC3 and AU3 codons. They show, that these bias is particular strong in larger mammals than other species. Interestingly, AU3 codons are more frequently used in GP1 genes, while the GC3 codons are more used to coding genes in GP2. These groups are associated with proliferation and differentiation, respectively. This is in agreement with previous finding, ref 13, who analysed tRNA abundance during these complementary programs. They also found that the GC3 content correlates with the abundance of transcript and found that this correlation can be a good tool to identify imbalance in the proliferation and differentiation processes, indicating diseases states. I found the work interesting and it provide novel results of interest to the field. However in the present form there still a number of points that must be corrected and/or clarified before deserve to be published.

We thank the reviewer for the comments.

Although the methods used are adequate, they are not clearly explained, on the other hand I believe that one of the secondary conclusions (codon employment shift is causal or effect) is not supported by the data. I encourage the author to correct the points above and re-submit the manuscript.

We are grateful for the useful comments that have helped us to make our manuscript more accurate. We believe that with the answers that we are providing in this letter and with the attached revised manuscript we have corrected and/or clarified the issues that were raised.

Abstract, line 32-32: "We found that abundant transcripts typically employed a subset of codons, while other codons were mostly present in low-abundance transcripts."
I could not connect this sentence with any part of the main text. Please explain to which are referring.

We refer specifically to the analysis that we explain on the updated manuscript on page 8 (line 215), where we define (and measure) the codon employment coefficient (CEC). The basic meaning of the CEC is that there is a correlation between mRNA abundance and percentage of certain codons in the mRNA. In detail, as we write in the main text: "We calculated for each codon the Pearson correlation coefficient between two vectors: 1) the % of the particular codon in the composition of each transcript (for example, the AAA codon, which encodes lysine, makes up between 0 and 18% of human transcripts, averaging at ~2.5%), and 2) the abundance of each transcript. In simple terms, this measure reflects how much a codon correlates to the mRNA abundances in a specific dataset".

To avoid misunderstandings, we have in part rephrased the sentence in the abstract: "We found that a subset of codons was preferentially employed in abundant transcripts, while other codons were preferentially found in low-abundance transcripts".

At the same time, we agree with the reviewer that this concept is not intuitive, so we have also phrased it more clearly in the main text (at page 8, lines 219-223): "Inherently these data demonstrate that a subset of codons was preferentially employed in abundant transcripts (those

with the most positive CEC values), while other codons were preferentially found in low-abundance transcripts (those with the most negative CEC values)".

Page2, line 23-24: "To our knowledge, this idea is virtually unexplored for human disease."
ref 14 is one case, moreover the authors could cite these papers:
1.- Synonymous Mutations Frequently Act as Driver Mutations in Human Cancers
10.1016/j.cell.2014.01.051
2.- Bicodon bias can determine the role of synonymous SNPs in human diseases
10.1186/s12864-017-3609-6

We have rephrased the sentence at page 3 (lines 74-75) and added the suggested references.

I do not understand figure 3A, which false discovery rate? Please explain better how you done this rank.

The false discovery rate (FDR) represented in **Fig. 3A** arises from the "Gene Ontology" term enrichment analysis. This technique allows to interpret sets of genes making use of the "Gene Ontology" system of classification, in which genes are assigned to a set of predefined groups depending on their functional characteristics. There are two ways of performing this analysis. The simplest one is to select a number of significantly enriched genes and score them against the whole reference gene set. The more complex one (but also more accurate and less biased), is to first rank genes for a specific measure (*e.g.* their relative expression) and then perform a "Gene Set Enrichment Analysis", as we have done in **Fig. 3**.

We agree with the reviewer that the legend of **Fig. 3A** in our original manuscript was in part cryptic (due to the word limit for the legends, since only 300 words are allowed). In any case, we have followed the suggestion of the reviewer and revised the legend to be clearer.

We have also explained in greater detail how the ranking was done and how the FDR was calculated in the updated "Methods" section:

"For the gene ontology (GO) enrichment analysis introduced in **Fig. 3** we used the WebGestalt 2017 gene set enrichment analysis toolkit (Wang et al., 2017). In detail we first identified the transcripts corresponding to all reviewed human proteins form the Uniprot database (UniProt Consortium, 2015). This allowed us to avoid the influence from badly GO-annotated transcripts (whose proteins are also not reviewed). This selection included 19'007 transcripts (mRNAs) for which we calculated the GC3 content. We than normalized the GC3 content by the average GC3 content of all these transcripts (58.30) and we calculated the $\log_2$ ratio for all these transcripts with respect to this average. We then used the id of each transcript and the $\log_2$ ratio as an input for the Gene Set Enrichment Analysis (GSEA) on WebGestalt looking into the functional database:

"Geneontology>Biological_Process_noRedundant"

This analysis revealed more than 60 GO identities whose false discovery rate (FDR) was significant (< 0.01), as detailed in **Additional File 1**. The FDR was obtained in the GSEA analysis through the default mode (BH) and thus calculated by the software with the Benjamini and Hochberg approach (Benjamini, Y. and Hochberg, 1995)."

It is not clear how authors classify the GP1 and GP2 genes in Table S2, why 600 and not 6 or 60 genes? Are there some threshold? please explain.

The reviewer is right, this point might not be easy to understand and it requires additional explanations, which we now provide in the updated version of the text at page 6 (lines 154-163). The genes that are represented in Supplementary Table 2 (renamed **Additional File 2** in the updated manuscript) are those that are significantly enriched in the ranked gene ontology analysis performed in **Fig. 3**. In fact, from this analysis we obtain both the significantly enriched GO categories, and the genes associated to these categories. These “genes associated to the significantly enriched GO categories” are represented in Supplementary Table 2 (now **Additional File 2**).

legend of fig 3, line 9: "The two groups of genes are connected with opposite biological processes. GP1 genes are important for cell division and cell cycling, while GP2 genes mediate cell differentiation and functions that arise in specialized organs. " I understand the sense but I believe that is no appropriate to talk about opposite biological processes in this case.

We have followed the suggestion of the reviewer and we have used a different wording: “completely different” instead of “opposite”.

I feel that figure 4 is not necessary for support results and/or conclusion (also for the lines 47-56 in page 5), fig 3 is enough, could you take out it or justify its presence?

Briefly, we believe that **Fig. 4** is important to show that the separation between GP1 and GP2 genes is not casual or stochastic, and because it provides a graphic representation of the GO categories that the average reader would appreciate (due to the quite peculiar division of the GO categories significantly enriched in GP1 and GP2 groups).

At the same time, we agree with the reviewer that the importance of this figure was not explained in sufficient detail in the original figure legend and in the text.

More in detail, this figure serves three functions: 1) It provides a graphic representation of the different processes (with relative sizes of each GO category); 2) It shows that GP1 and GP2 gene ontology categories are linked within each of the two groups by a higher number of interconnections than across the two groups. This suggests that within GP1 and GP2 there are similarities. 3) As detailed in the main test, upon randomization of the genes assigned to the two groups, there is a strong (~26 fold) increase of number of interconnections between the two groups, implying that the separation of the two groups of genes is not stochastic and likely follows a rational organization.

To more appropriately justify the presence of this figure, we have added these concepts in the legend and we have also included a sentence in the conclusions at the end of the paragraph (page 7, lines 191-193 of the revised manuscript).

In any case, if the reviewer feels particularly strong about removing this figure from the main text we would be willing of moving it to the supplement containing the additional files.

corrCEC is not well defined:
"The codon composition of each transcript, in %, was determined (consisting of 61 codon percentages). This was correlated to the codon CECs in controls and in disease samples. "
could the authors specify more clearly which vectors are correlated?

To understand the corrCEC it is necessary to first define the codon employment coefficient (CEC, **Fig. 5**). The CEC was calculated as follows. For each codon we determined the % it represents of the composition of each transcript. We then correlated this set of values with the abundance of the transcripts. The resulting Pearson's correlation coefficient represents the CEC. In simple terms, this measure reflects how much a codon correlates to the mRNA abundances in a specific dataset. As an example, if a codon makes up a high percentage of the composition of the most abundant mRNAs, its CEC will be high, while if it is used more often in the least abundant mRNA its CEC will be low (negative). Inherently these data demonstrate that a subset of codons was preferentially employed in abundant transcripts (those with the most positive CEC values), while other codons were preferentially found in low-abundance transcripts (those with the most negative CEC values).

The "correlation of transcript composition to codon employment", or CorrCEC (**Figures 7-8**), was determined as follows. The codon composition of each transcript, in %, was again determined (consisting of 61 codon percentages). The codon composition was correlated to the codon CECs in controls and in disease samples, for every single transcript. In simple terms, this verifies whether the composition of the respective transcript more closely correlates to the preferred codon employment in disease or in the control situation. The difference between the two resulting correlation coefficients was termed the CorrCEC (where negative values indicate a correlation to the preferred codon employment in the control situation, while positive values show a correlation to the disease situation). Overall, the CorrCEC can be used to pinpoint genes whose composition (in terms of codons) mirrors the codon usage in disease or in the control situation.

We have added these explanations to the updated main text and Methods.

The subsection "The shift in codon employment appears to drive the transcript abundance changes, rather than being a consequence of these changes" is not to clear for me. Codon employment shift is computed from CEC which in turn is defined by the codon usage and mRNA abundances. the shift is due to abundance because you are using the same sequences. How you can conclude the inverse case? Fig S2 could be moved and discussed better in the main text.

We thank the reviewer for this comment. The point is indeed a complex one. To clarify it we moved the figure to the main text as suggested by the reviewer. We explain this point here and we have added an analogous clarification in the main text:

If the abundance of a number of transcripts that are particularly rich in, for example, AU3 codons, rises profoundly, then the CEC is pushed in the respective direction, as the reviewer noticed. In this case a simple consequence would be that, on average, the abundance of the transcripts correlates to their levels of AU3 codons, simply because the highly abundant codons happen to be AU3-rich. But, if one is to selectively analyze AU3-rich transcripts, there is no *a priori* expectation that their abundances correlate to the AU3 levels. In other words, if they are just expressed independent of each other, there is no particular reason for which very AU3-rich transcripts should be more abundant than moderately AU3-rich transcripts. Conversely, if we analyze only AU3-poor transcripts (GC3-rich ones), there is no reason why very AU3-poor transcripts should have lower abundances than moderately AU3-poor ones.

If, however, the codon employment shift is causal in nature, and the organism has switched it to the AU3 direction, then all transcripts are affected. Very AU3-rich transcripts will be more abundant than moderately AU3-rich constructs, and very AU3-poor transcripts will be less abundant than moderately AU3-poor transcripts. This is the situation we found in human tissues, and this strongly suggests a causal nature for this phenomenon."

The specificity in figure 8 refer to the same group of sample, the method could no be able to discriminate between different diseases as discussed in page 13 of conclusion. Could the authors explained the limitations of the term "specificity" in the context of the figure 8?

The term “specificity” in Figure 8 is only used in the context of medical diagnosis (and statistics), where the “sensitivity” is the ability of a test to correctly identify patients with the disease (true positive rate), whereas the “specificity” is the ability of the test to correctly identify those without the disease (true negative rate). We have detailed this concept in the legend of **Fig. 9** at page 30 (lines 800-802) of our revised manuscript to avoid misunderstandings. As the reviewer correctly noticed, we are openly admitting in the conclusions the fact that this method might not be sufficient to discriminate different pathologies. As we openly discuss, in order to test this possibility, one would need to have access to large multi-disease studies. While we believe that this might be a future development of this line of research we are convinced that this point exceeds the scope of our current work.

There are several typos in the text, like in line 27 page 12 "is has" and "emloyment shift" in the axes label of figure S2.

We thank the reviewer for spotting the typos, which are now corrected in the updated version of the manuscript.

References:

Benjamini, Y. and Hochberg, Y. (1995). Controlling the False Discovery Rate : A Practical and Powerful Approach to Multiple Testing Author (s): Yoav Benjamini and Yosef Hochberg Source : Journal of the Royal Statistical Society . Series B (Methodological), Vol . 57 , No . 1 Published by : J. R. Stat. Soc. B *57*, 289–300.

UniProt Consortium (2015). UniProt: a hub for protein information. Nucleic Acids Res. *43*, D204-12.

Wang, J., Vasaikar, S., Shi, Z., Greer, M., and Zhang, B. (2017). WebGestalt 2017: a more comprehensive, powerful, flexible and interactive gene set enrichment analysis toolkit. Nucleic Acids Res. *45*, 1–8.

Reviewer 2:

The manuscript entitled "Pathological changes are associated with shifts in the employment of synonymous codons at the transcriptome level" is well-written. The figures and tables are informative and helpful. The paper is very interesting and unique in its work. However, I have a few minor concerns regarding this article.

We thank the Reviewer for appreciating our work.

1. The abstract may be revised as per BMC Genomics guidelines i.e. sub heading of abstract must include background, results and conclusions.

We have revised the abstract as requested.

2. In page number 2 and line number 6, reference style should be uniform throughout the manuscript.

We have homogenized the reference style following the guidelines from BMC.

3. The scientific name should be italicized, for example Drosophila in page number 2 and line number 51.

We have italicized the scientific names as requested.

4. Relative synonymous codon usage should be added to elucidate the over-represented and under-represented codons for GC3-rich and AT3-rich genes.

We thank the reviewer for the suggestion. We have added the relative synonymous codon usage figure for the GP1 and the GP2 genes as requested. This analysis is included in the updated manuscript as **Additional File 3** (**Supplementary Figure 1**). As expected, the relative synonymous codon usage confirmed that GP1 transcripts prefer AU3 codons, while GP2 have a preference for GC3 codons.

Reviewer 3:

This is an excellent article, very well written.
The background is solid, the methods are appropriately described, and the results are adequately presented and discussed.
The findings are relevant for the field of human genomics and of great interest for the readers of this journal.
I have only some minor comments.

We thank the Reviewer for appreciating our work

1. Page 1. "A- and U-ending codons" should be corrected in "A- or U-ending codons".

We have corrected the sentence as suggested

2. Page 6. The concept here named by the Authors as "Codon employment coefficient" appears to be analogous to the "codonome bias" concept proposed by Piovesan et al., Genomics, 2013. This should be recalled here, or in the Discussion, also considering that an original software with graphical interface to perform this type of calculations was made available there, and that RNA abundance to feed it was calculated by an approach allowing cross-platform expression data integration.

We thank the Reviewer for bringing this important reference to our attention, which we have added on page 8 (lines 221-223) as suggested.

3. Page 12. "GC3/AU3 bias is has a role" should be corrected in "GC3/AU3 bias has a role".

We have corrected the typo.

4. Table_S4.xlsx. Adding a column with the name of the experimental platform of analysis used for each GEO series would be useful here.

We have added the platform used for the experiments in **Supplementary Table 4** (now renamed **Additional File 7**) as requested.
